# Separation of Temperature-Induced Response for Bridge Long-Term Monitoring Data Using Local Outlier Correction and Savitzky–Golay Convolution Smoothing

**DOI:** 10.3390/s23052632

**Published:** 2023-02-27

**Authors:** Wei Zhang, Hongyin Yang, Hongyou Cao, Xiucheng Zhang, Aixin Zhang, Nanhao Wu, Zhangjun Liu

**Affiliations:** 1 School of Civil Engineering and Architecture, Wuhan Institute of Technology, Wuhan 430073, China; 2 Hubei Provincial Engineering Research Center for Green Civil Engineering Materials and Structures, Wuhan 430073, China; 3 School of Civil Engineering and Architecture, Wuhan University of Technology, Wuhan 430070, China; 4 Engineering Research Center of Disaster Prevention and Mitigation of Southeast Coastal Engineering Structures (JDGC03), Fujian Province University, Putian 351100, China

**Keywords:** structural health monitoring, bridge, temperature-induced response, signal separation, local outlier factor, optimization algorithm

## Abstract

This study proposed a separation method to identify the temperature-induced response from the long-term monitoring data with noise and other action-induced effects. In the proposed method, the original measured data are transformed using the local outlier factor (LOF), and the threshold of the LOF is determined by minimizing the variance of the modified data. The Savitzky–Golay convolution smoothing is also utilized to filter the noise of the modified data. Furthermore, this study proposes an optimization algorithm, namely the AOHHO, which hybridizes the Aquila Optimizer (AO) and the Harris Hawks Optimization (HHO) to identify the optimal value of the threshold of the LOF. The AOHHO employs the exploration ability of the AO and the exploitation ability of the HHO. Four benchmark functions illustrate that the proposed AOHHO owns a stronger search ability than the other four metaheuristic algorithms. A numerical example and in situ measured data are utilized to evaluate the performances of the proposed separation method. The results show that the separation accuracy of the proposed method is better than the wavelet-based method and is based on machine learning methods in different time windows. The maximum separation errors of the two methods are about 2.2 times and 5.1 times that of the proposed method, respectively.

## 1. Introduction

Large-scale infrastructure is affected by various factors in the process of long-term use, such as material aging, climatic conditions, and external forces action. These factors will accelerate the damage accumulation and resistance deterioration of the structure and even cause a collapse in severe cases [1,2]. Structural health monitoring (SHM) technology is critical for ensuring structural reliability and safety. Real-time SHM monitoring data can be used to invert the internal force changes in the structure. Furthermore, it provides a reference and basis for the structural damage location, safety analysis, and evaluation [3].

With the advent of the digital age, SHM systems are increasingly used in large-scale civil buildings [4,5]. In the process of a bridge operation, many factors, such as live loads, temperature actions, and concrete shrinkage and creep, will affect the measured response of the structure [6]. A key component of the bridge response is the temperature-induced response, which can sometimes exceed the live load-induced response [7,8,9]. The failure to correctly simulate temperature-induced responses may lead to undetectable structural damage or even obtain wrong results [10]. Therefore, the temperature-induced response should be distinguished from the monitoring data for a structural damage diagnosis and safety warning [11,12].

The mapping relationship between the temperature and the structural response is used to separate the temperature-induced response in most of the previous publications [13,14]. For instance, Sun et al. [15] established a linear regression model to separate structural temperature-induced responses. Nevertheless, the fixed regression model cannot provide accurate long-term predictions because the relationship between the structural response and temperature is dynamic [9,16]. Moreover, for a complete structure, regression-based algorithms need to be trained with measurements for at least one year [17]. To address the limitations of the fixed regression model, it is a superior separation approach to construct the bridge-temperature model based on the probability connection using a Bayesian inference which is an effective quantitative method for uncertainty. Huang et al. [18] used the sparse Bayesian learning theory to establish a structural temperature response prediction algorithm that can quantitatively reflect the temperature deformation characteristics of bearings. Subsequently, Wang et al. [19] proposed a new Bayesian model for predicting the temperature-induced response of a cable-stayed bridge. In the prediction performance, the new model outperforms the autoregressive, multivariate linear regression, and Bayesian dynamic linear models without autoregressive components.

Information about the temperature distribution of the environment or structure needs to be provided for the separation method of establishing the mapping relationship between the temperature and structural response. However, it is sometimes inconvenient or impossible to obtain the information. Hence, using single-response data, the temperature-induced response is directly separated from the structural response, which will expand the range of practical applications [20,21]. A multi-resolution wavelet has been widely used in structural temperature-induced response separation due to its outstanding characteristics in processing instantaneous and non-stationary signals in the time-frequency domain. Many existing studies use multi-resolution wavelets to separate the temperature-induced response from the original signal [22,23]. For instance, Xu et al. [24] proposed a multi-resolution wavelet analysis method based on the frequency bandwidth. According to the ability of the decomposition layer to divide the overall signal bandwidth, the theoretical formula for determining the decomposition layer is derived, and the temperature-induced response from the bridge response is revealed. Ye et al. [25] and Zhu et al. [26] proposed a comprehensive machine learning algorithm, combining the ensemble empirical pattern decomposition, principal component analysis, and fast independent component analysis (EEPI). The temperature impacts on bridge responses are revealed using the method. However, the separation accuracy of the machine learning algorithm is greatly affected by noise. Furthermore, the outcomes of these methods are susceptible to some critical parameters. 

As information and data processing technologies have advanced, intelligent optimization algorithms have become more competitive in interpreting temperature-induced responses. Optimal critical parameters are obtained using intelligent optimization algorithms to achieve an optimal separation or predictive response. Liu et al. [27] proposed an adaptive filtering method for separating temperature-induced responses by using the PSO algorithm based on the characteristics that daily temperature-induced responses are independent of other reactions on the time scale. Fei et al. [28] proposed an innovative algorithm that combines the Jaya optimizer and Salp group algorithm to forecast the long-term temperature-induced response. Xu et al. [29] proposed a practical approach for separating the temperature-induced responses of long-span bridges based on a first-order optimization algorithm. The approach improves the computation efficiency using a multivariate linear model and discrete temperature actions. The support vector machine is an effective method for processing time-series prediction models. However, for complex models, the effect of the support vector machine regression model is not ideal. Therefore, deep learning models and multi-layer neural network structures are usually combined in such models [30]. For instance, Yue et al. [31] suggested a novel digital regression model based on deep learning to separate the temperature-induced deflection. The results reveal that the accuracy and robustness of the method are superior to conventional regression models. Long and short neural networks can process complex time-series data and make accurate predictions, so they are suitable for forecasting in various situations [32].

The above studies show that the structural temperature-induced response separation usually uses a multi-scale analysis to decompose the total response of the structure [33]. Generally, there are two types of separation methods: the model-based and signal-processing methods. The model-based approach relies on the accuracy of the FEM. In general, the initial FEM should be updated. Then, the temperature-induced response is separated and predicted according to the mapping relationship between the temperature and structural response [19,34]. However, establishing a reliable FEM that can describe the inherent structural characteristics is a great challenge [35]. Moreover, sometimes the structural temperature distribution cannot be obtained, which limits the application space of this method. The way based on signal processing does not need to understand the structural physical characteristics. The signal is usually decomposed according to the range of different frequency domains to separate the temperature-induced response. However, the separation accuracy of the way based on signal processing is usually affected by various factors, such as the signal bandwidth and mode aliasing. Moreover, some of these methods are greatly affected by noise.

This study focuses primarily on the separation performance of the accuracy and anti-noise. The proposed method fully uses the characteristics that the monitoring data will change sharply in a short time (data mutation) when the bridge structure is affected by live loads. The AOHHO algorithm determines the optimal critical parameters. The mutational data are corrected and smoothed to obtain a temperature-induced response. Then, the method is compared to two other conventional methods for evaluating its separation performance. Finally, the method is used to analyze the correlation between the field-effective temperature and separated deflection.

## 2. Methodology

### 2.1. Separation of Temperature-Induced Response

#### 2.1.1. Components of Bridge Response

The measured deflection usually contains several types of responses, such as live load, temperature, irreversible influencing factors, and noise-induced responses [36]. Therefore, the bridge response can be expressed as

(1) $$D=D_{L}+D_{T}+D_{E}+D_{I}$$

where $D_{L}$, $D_{T}$, and $D_{E}$ are the response caused by live loads, temperature, and noise, respectively; $D_{I}$ is irreversible structural response, which is caused by creep, shrinkage, and prestress loss.

#### 2.1.2. Local Outlier Factor

The internal force and deformation of the bridge will change dramatically due to the action of live loads [37,38]. Accordingly, the monitoring data will mutate. Therefore, it is possible to eliminate the live loads-induced response using this characteristic.

The local outlier factor is used to judge whether there is a mutation in the discrete data. Then, mutational data will be corrected [39]. Its purpose is to eliminate the response caused by live loads in the structural response. The LOF algorithm compares the density of each point to the density of its surrounding points to determine whether it is an outlier. The calculation of LOF is divided into four steps as follows:

- Step 1: *k*-distance and its neighborhood

The *k*-distance of a point *x* represents the length between *x* and a point *o,* which is the *k*th-nearest point to *x*. The *k*-distance can be expressed as

(2) $$d_{k}\left(x\right)=d\left(x,o\right)\;\;\;\left(x\notin M,\;o\in M\right)$$

where *M* represents a set, and *o* must meet the following requirements:

(i) Up to *k* − 1 points a∈M such that d(x,o)>d(x,a);
(ii) At least *k* points a∈M such that d(x,o)≥d(x,a).

**Figure 1:**
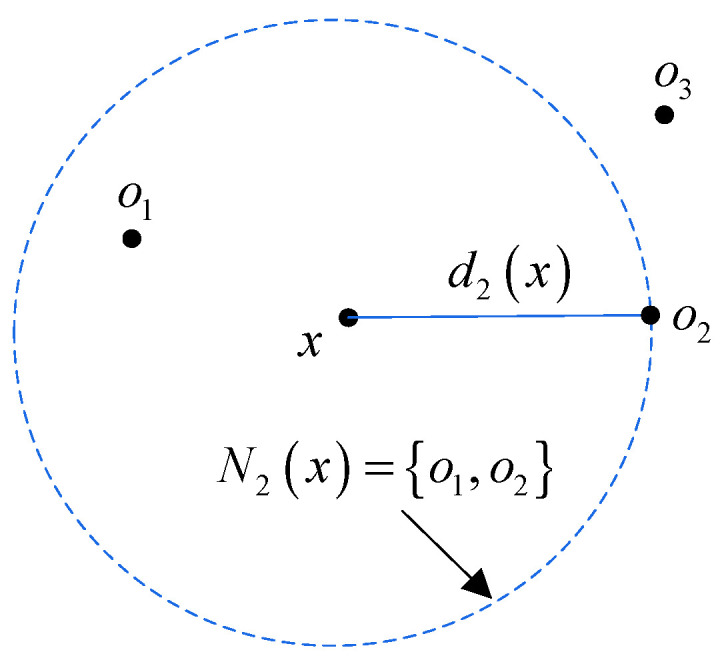
Illustration of *k*-distance and its neighborhood of the point *x*(*k* = 2).

The *k*-distance neighborhood of *x* represents a set, denoted as $N_{k}\left(x\right)$, and all points *o* in the set are no more than $d_{k}\left(x\right)$ away from *x*. Figure 1 visually explains *k*-distance and its neighborhood.

b. Step 2: reachability distance

The reachability distance from the point *x* and the point *o*, denoted as $Rd_{k}\left(x,o\right)$, is the larger of their actual distance and *k*-distance. The reachability distance can be expressed as

(3) $$Rd_{k}\left(x,o\right)=\max\left\{d\left(x,o\right),d_{k}\left(x\right)\right\}$$

Equation (3) shows that the minimum value of the reachability distance is at least $d_{k}\left(x\right)$. For example, it holds that $d_{2}\left(x\right)=Rd_{2}\left(x,o_{1}\right)=Rd_{2}\left(x,o_{2}\right)<Rd_{2}\left(x,o_{3}\right)=d\left(x,o_{3}\right)$ in Figure 1.

c. Step 3: local reachability density

The local reachable density, whose definition is related to reachability distance, is an absolute density, and it can be calculated using Equation (4).

(4) $$Ld_{k}\left(x\right)={\left(\frac{\sum_{o\in N_{k}\left(x\right)}^{}Rd_{k}\left(x,o\right)}{\left|N_{k}\left(x\right)\right|}\right)}^{-1}$$

where $\left|N_{k}\left(x\right)\right|$ is the number of elements in set $N_{k}\left(x\right)$.

d. Step 4: local outlier factor

The local outlier factor $LOF_{k}\left(x\right)$ is a relative density, represents the degree of outliers of a point *x*. It can be expressed as

(5) $$LOF_{k}\left(x\right)=\frac{\sum_{o\in N_{k}\left(x\right)}^{}Ld_{k}\left(o\right)}{\left|N_{k}\left(x\right)\right|\times Ld_{k}\left(x\right)}$$

In summary, if a point *x* is distant from other points *o*, its local reachable density is obviously tiny. The LOF algorithm measures the anomaly degree of a point *x*, not based on its absolute density but on its relative density concerning its neighboring point *o*. Therefore, the LOF algorithm can be applied to uneven data distributions with different densities.

#### 2.1.3. Adaptive Method Based on Local Outlier Correction and Smoothing

The bridge response varies with the number, type, and speed of vehicles crossing the bridge. Hence, the mutational degree of measured data is fluctuant. Moreover, the amount of monitoring data, which will rise or fall as a whole for an extended period, is enormous. A better separation result cannot be achieved if a fixed judgment standard is used to deal with it. Therefore, splitting the original data sequence into several subsets is necessary, setting different thresholds to process them.

The original data sequence X is sequentially divided into *N* subsets. $X_{g}=\left\{x_{1}^{g},x_{2}^{g},...,x_{v}^{g}\right\}$ denotes the *g* th subset of X, and each subset corresponds to a threshold $\varepsilon_{k}^{g}$. If the $LOF_{k}\left(x_{i}^{g}\right)$ exceeds $\varepsilon_{k}^{g}$, it is considered to be affected by live loads, and a new data sequence $Y_{g}=\left\{y_{1}^{g},y_{2}^{g},...,y_{v}^{g}\right\}$ is obtained by correcting the original data. $Y_{g}$ is mainly a temperature-induced response with noise. The specific mathematical expression is defined as

(6) $$y_{i}^{g}=\{\begin{array}{ll} x_{i-1}^{g} & \;if\;LOF_{k}\left(x_{i}^{g}\right)>\varepsilon_{k}^{g}\\ x_{i}^{g} & \;if\;LOF_{k}\left(x_{i}^{g}\right)\leq \varepsilon_{k}^{g} \end{array}$$

where $\varepsilon_{k}^{g}$ is the threshold of the *g* th subset of X.

**Figure 2:**
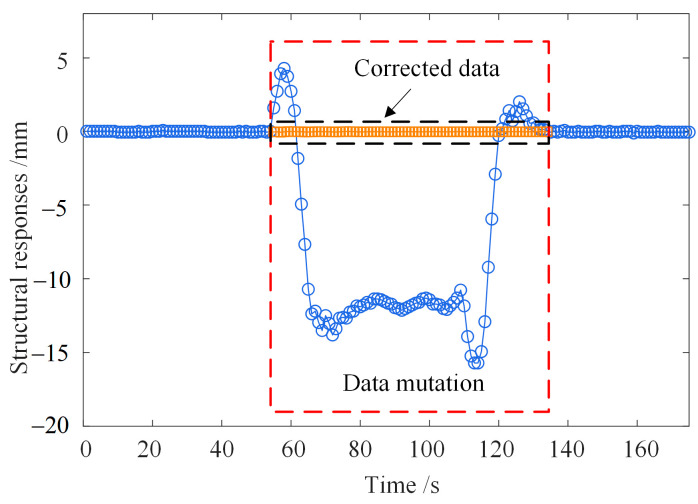
Comparison of structural responses before and after correction.

Figure 2 shows the comparison of structural responses before and after correction. 

**Figure 3:**
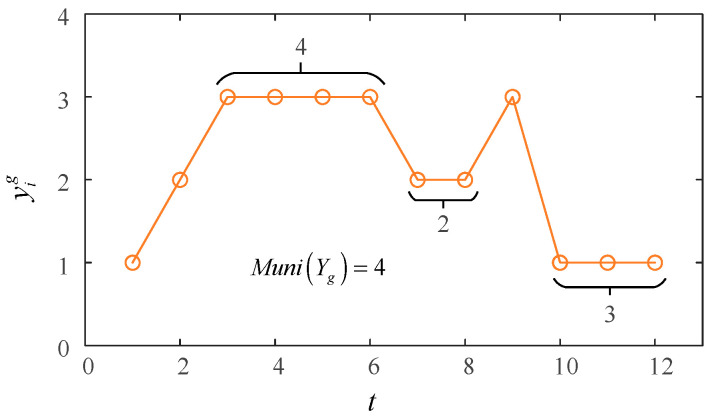
Illustration of $Muni\left(Y_{g}\right)$.

Equation (6) shows that setting a reasonable threshold $\varepsilon_{k}^{g}$ is one of the critical problems for separating the temperature-induced response. If the setting $\varepsilon_{k}^{g}$ is too small, it will cover the actual trend of structural response. If the setting $\varepsilon_{k}^{g}$ is too large, it will cause significant fluctuations in the monitoring data, which cannot achieve a better separation effect. Therefore, the setting $\varepsilon_{k}^{g}$ can be transformed into an optimization problem with constraints. Its objective function and constraints are defined as

(7) $$obj=\min\left(\frac{{\sum_{i=1}^{v}\left(y_{i}^{g}\left(\varepsilon_{k}^{g}\right)-{\bar{y}}_{i}^{g}\left(\varepsilon_{k}^{g}\right)\right)}^{2}}{v}\right)$$

(8) $$s.t.\;\;\{\begin{array}{c} a\leq \varepsilon_{k}^{g}\leq b\\ Muni\left(Y_{g}\right)\leq Q \end{array}$$

where $Muni\left(Y_{g}\right)$ is the maximum of the number of continuous equal data in the subset $Y_{g}$, as shown in Figure 3; *Q* is a constant value determined by the number of data; *a* and *b* are constants of 1 and 2.5, respectively.

After adaptive local outlier correction, *Y*_g_ usually contains some noise signals. Savitzky–Golay convolution smoothing (S-G smoothing) is widely used to eliminate noise, a filtering method based on polynomial fitting [40]. The essential feature is that the width and shape of the signal can remain unchanged in the filtering process. Therefore, the temperature-induced response can be obtained using S-G smoothing to filter out the noise in *Y*_g_. The mathematical expression is defined as

(9) $$D_{S}=Smo\left(Y_{g}\right)$$

where $D_{S}$ is the separated temperature-induced response, and Smo(·) represents S-G smoothing.

### 2.2. AOHHO Algorithm

As described in Section 2.1.3, setting a reasonable $\varepsilon_{k}^{g}$ is one of the critical issues for separating temperature-induced responses. The setting $\varepsilon_{k}^{g}$ can be transformed into an optimization problem with constraints. Therefore, an AOHHO hybrid optimization algorithm is proposed, derived from the Aquila Optimizer (AO) [41] and Harris Hawks Optimization (HHO) [42] algorithms with similar structures.

In the AOHHO algorithm, the location of each individual represents a feasible solution. The way of location update represents a search process for feasible solutions. The AOHHO algorithm consists of two phases, exploration steps and exploitation steps, and can find the optimal solution using different equations according to different conditions. The calculation model of AOHHO is divided into steps as follows.

#### 2.2.1. Exploration Phase

- Step 1

In this step, the location update mode of the individual can be expressed as

(10) $$X(t+1)=X_{m}(t)-X_{b}(t)\times R+X_{b}(t)\times \left(1-\frac{t}{T}\right)$$

where $X_{m}(t)$ represents the mean of all solutions in the current iteration; $X_{b}(t)$ is the current optimal solution; *R* is a random value inside (0, 1); t is the current iterative number; T is the maximum iterative number.

b. Step 2

In this step, the individual adopts Equation (11) to update the location.

(11) $$X(t+1)=X_{R}(t)+R\times (x-y)+X_{b}(t)\times Levy(D)$$

where *D* is the dimension space of variables, $X_{R}(t)$ represents random location of the individual, Levy(D) is calculated using Equation (12).

(12) $$Levy(D)=s\times \frac{u\times \sigma }{|v{|}^{\frac{1}{\beta }}}$$

(13) $$\sigma =\left(\frac{\Gamma (1+\beta )\times sin\left(\frac{\pi \beta }{2}\right)}{\Gamma \left(\frac{1+\beta }{2}\right)\times \beta \times 2^{\left(\frac{\beta -1}{2}\right)}}\right)$$

where β and s are fixed values of 1.5 and 0.01, respectively; *v* and *u* are random values inside (0,1); y and x are calculated in Equation (14).

(14) $$\{\begin{array}{c} x=\left(r_{1}+U\times D_{1}\right)\times \cos(\theta )\\ y=\left(r_{1}+U\times D_{1}\right)\times \sin(\theta )\\ \theta =-\omega \times D_{1}+\frac{3\times \pi }{2} \end{array}$$

where $r_{1}$ is a random number inside (1, 20), U is constant equal to 0.00565, $D_{1}$ is an integer inside (1, *D*), and ω is equal to 0.005.

#### 2.2.2. Transition Mechanism

In this step, the transition from exploration steps to exploitation steps is achieved by utilizing the escaping energy of the individual. The escaping energy of the individual can be simulated as

(15) $$E=2E_{0}\times \left(1-\frac{t}{T}\right)$$

where $E_{0}$ represents the initial energy.

#### 2.2.3. Exploitation Phase

- Step 1

In this step, the individual location update is simulated by Equation (16).

(16) $$X(t+1)=X_{b}\left(t\right)-X\left(t\right)-E\times \left|X\left(t\right)-JX_{b}\left(t\right)\right|$$

(17) J=2×(1−R)

where J is the random jumping intensity of the individual.

b. Step 2

In this step, the individual adopts Equation (18) to update the location.

(18) $$X(t+1)=X_{b}\left(t\right)-E\times \left|X\left(t\right)-X_{b}\left(t\right)\right|$$

c. Step 3

In this step, the individual location update is simulated by Equation (19).

(19) $$X(t+1)=\{\begin{array}{ll} Y_{1} & \;if\;F\left(X\left(t\right)\right)>F\left(Y_{1}\right)\\ Z_{1} & \;if\;F\left(X\left(t\right)\right)>F\left(Z_{1}\right) \end{array}$$

(20) $$Y_{1}=X_{b}\left(t\right)-E\times \left|X\left(t\right)-JX_{b}\left(t\right)\right|$$

(21) $$Z_{1}=Y_{1}+S_{r}\times Levy(D)$$

where $S_{r}$ is a random vector.

d. Step 4

In this step, the individual adopts Equation (22) to update the location.

(22) $$X(t+1)=\{\begin{array}{ll} Y_{2} & \;if\;F\left(X\left(t\right)\right)>F\left(Y_{2}\right)\\ Z_{2} & \;if\;F\left(X\left(t\right)\right)>F\left(Z_{2}\right) \end{array}$$

(23) $$Y_{2}=X_{b}\left(t\right)-E\times \left|X_{m}\left(t\right)-JX_{b}\left(t\right)\right|$$

(24) $$Z_{2}=Y_{2}+S_{r}\times Levy(D)$$

#### 2.2.4. Performance Evaluation of AOHHO

**Figure 4:**
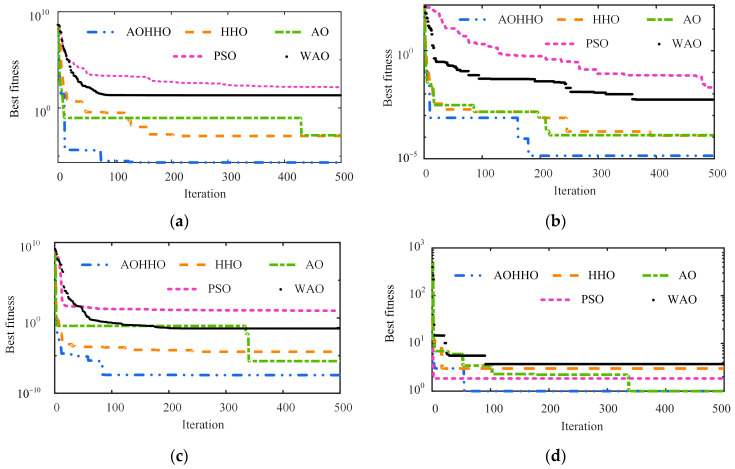
Iterative curves of each benchmark function: (**a**) F1; (**b**) F2; (**c**) F3; (**d**) F4.

The optimization potential of the AOHHO algorithm was examined using four typical test functions and compared with that of the AO, HHO, PSO, and WAO algorithms. The development potential of AOHHO is tested using the unimodal test benchmark functions F1 and F2. The multimodal benchmark function F3 is designed to assess the exploration trend of AOHHO. The fixed-dimensional multimodal benchmark F4 demonstrates the ability of AOHHO to explore space in low dimensions. The four benchmark functions are shown in Table 1 [41,42]. The number of iterations and population size in these algorithms are 500 and 30, respectively. The iterative curve corresponding to each function is shown in Figure 4. 

As shown in Figure 4, the convergence speed of the AOHHO algorithm is remarkably faster than other optimization algorithms, and its fitness is less than the four basic algorithms. The AOHHO algorithm utilizes the advantages of the AO and HHO algorithms, including global search capability, fast convergence speed, and development capability. Therefore, compared with the different optimization algorithms, AOHHO has significant advantages.

### 2.3. Flow Chart of the Proposed Method

**Figure 5:**
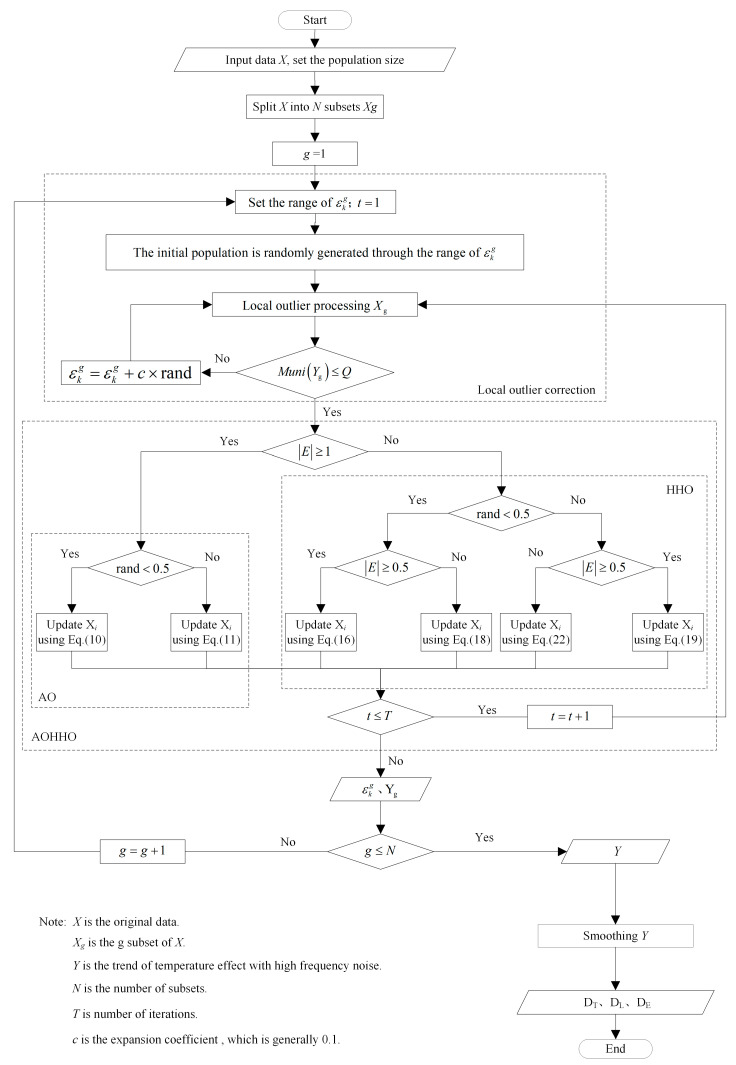
Flow chart of the proposed method.

The proposed method is divided into two main parts: local outlier correction and the AOHHO algorithm. The AOHHO algorithm is used to obtain the optimal threshold $\varepsilon_{k}^{g}$. The local outlier is then detected using the threshold $\varepsilon_{k}^{g}$. Next, the outliers are corrected to obtain the temperature-induced response with noise. Finally, the temperature-induced response can be obtained using S-G smoothing to filter out the noise in $Y_{g}$. Figure 5 depicts the flow chart for the proposed method.

## 3. Illustrative Examples

The case provided by the reference [24] is used to investigate the accuracy and anti-noise of the proposed method and compare it with the wavelet-based [24] and the EEPI-based [25,26] methods. The efficiency of the three approaches is discussed at per minute and hour scales. The expression of the simulated signal can be expressed as [24]

(25) $$D_{M}=D_{\mathrm{str}}+D_{\mathrm{dtr}}+D_{L}$$

(26) $$D_{\mathrm{str}}=A_{\mathrm{str}}\times \sin\left(\frac{\pi x}{1.5768\times 10^{7}f_{s}}\right)$$

(27) $$D_{\mathrm{dtr}}=A_{\mathrm{dtr}}\times \sin\left(\frac{\pi x}{4.32\times 10^{4}f_{s}}\right)$$

(28) $$D_{L}=\{\begin{array}{ll} N_{\mathrm{OR}}(1,i) & \;if\;N_{\mathrm{OR}}(1,i)\leq 5\\ 0 & \;if\;N_{\mathrm{OR}}(1,i)>5 \end{array},\;N_{\mathrm{OR}}\sim N\left(0,\sigma^{2}\right)$$

where $D_{\mathrm{str}}$ and $D_{\mathrm{dtr}}$ represent the seasonal and daily temperature-induced responses, respectively; $A_{\mathrm{str}}$ is a constant equal to 60; $A_{\mathrm{dtr}}$ is a random value inside (7, 20); $f_{s}$ is the frequency; σ is a fixed value of 10; $N_{\mathrm{OR}}$, a one-dimensional array, represents a normal distribution with a mean of 0.

(29) $$\delta =D_{S}-D_{\mathrm{str}}-D_{\mathrm{dtr}}$$

where δ is the separation error, $D_{S}$ is the separated temperature-induced response.

### 3.1. Discussion of Separation Accuracy

**Figure 6:**
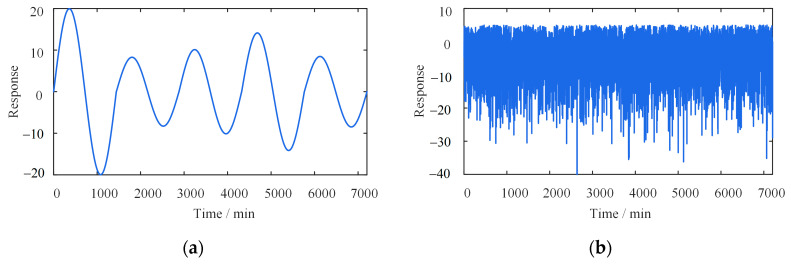

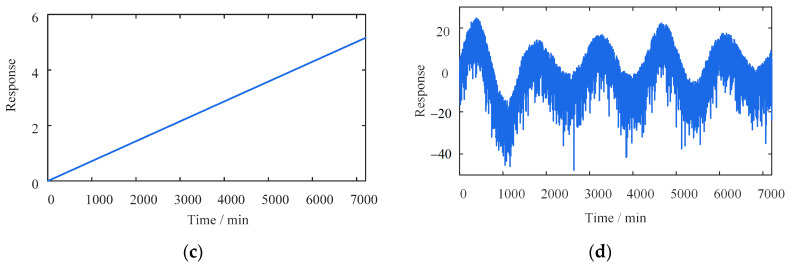
Different types of simulated signals (five days). (**a**) Daily temperature-induced response; (**b**) traffic-induced response; (**c**) seasonal temperature-induced response; (**d**) mixed signals.

**Figure 7:**
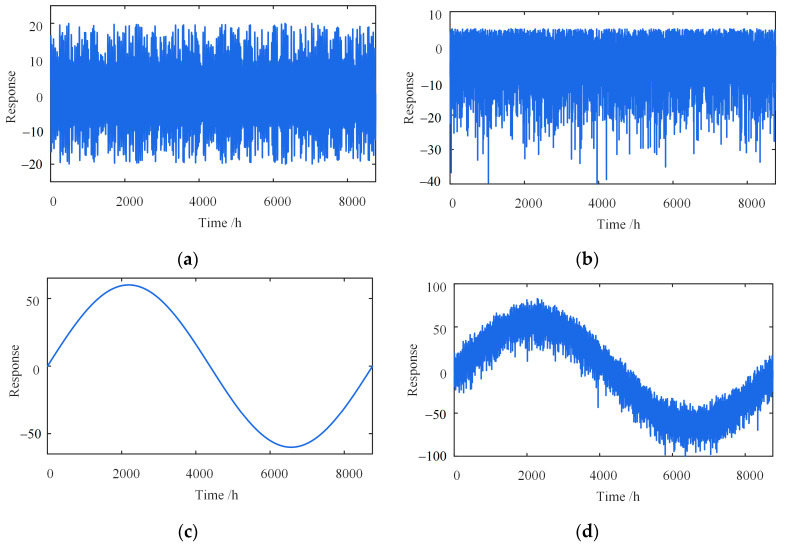
Different types of simulated signals (one year). (**a**) Daily temperature-induced response; (**b**) traffic-induced response; (**c**) seasonal temperature-induced response; (**d**) mixed signals.

Figure 6 and Figure 7 show various simulated signals in five days and one year, such as the daily temperature-induced response, traffic-induced response, seasonal temperature-induced response, and mixed signals. The scales of these two time windows are minutes and hours, respectively. In this case, every 60 points are a subset, and each subset corresponds to a threshold $\varepsilon_{k}^{g}$. The corresponding calculation parameters are shown in Table 2.

**Figure 8:**
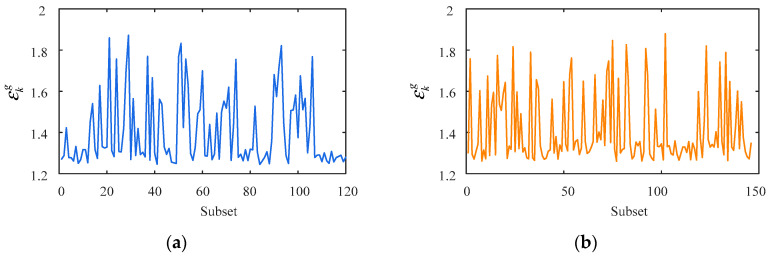
Curve of the optimal $\varepsilon_{k}^{g}$ of each subset in different time windows. (**a**) Five days; (**b**) one year.

Figure 8 shows the optimal $\varepsilon_{k}^{g}$ curve of each subset of the proposed method, with $\varepsilon_{k}^{g}$ fluctuating between 1.2 and 2.0. $LOF_{k}\left(x_{i}^{g}\right)$ is a density-based ratio. The closer the ratio is to 1, which indicates that the point is closer to other points in the neighborhood, the more likely it is normal. On the contrary, the greater the ratio is than 1, the more likely the point is an outlier.

**Figure 9:**
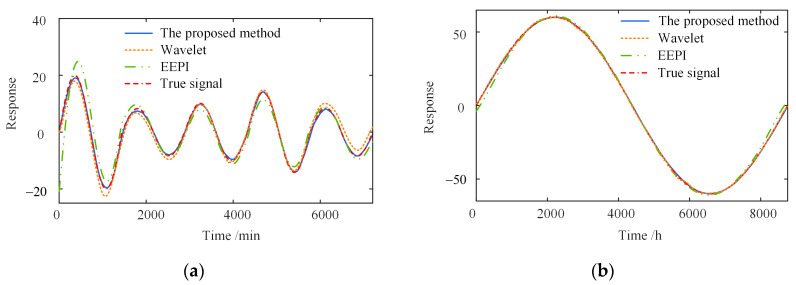
Comparison of separation results in different time windows. (**a**) Five days; (**b**) one year.

Figure 9 compares the separation results of the temperature-induced responses in different time windows. The separation results of the three methods are consistent with the actual signal in the trend. Moreover, the one-year separation result is more accurate in the amplitude than the five-day one. The significant errors of the wavelet-based and EEPI-based methods are concentrated on the peaks and troughs.

**Figure 10:**
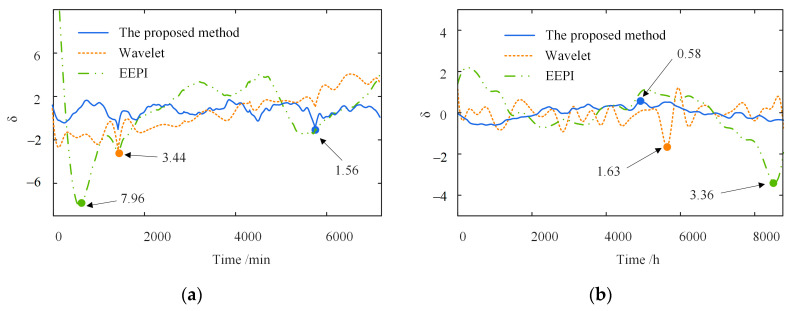
Separation error in different time windows. (**a**) Five days; (**b**) one year.

Figure 10 shows the separation error δ in the different time windows. In contrast to the other two methods, the proposed method has a much smaller separation error δ on the different time scales. The shorter the time window, the greater the superiority of the proposed method. Furthermore, as shown in Figure 10a, the maximum δ of the wavelet-based method is 3.44, about 2.2 times that of the proposed method. The maximum δ of the EEPI-based method is 7.96, without considering the error caused by the “end effect”, about 5.1 times that of the proposed method. Simultaneously, the separation error δ within five days is more extensive than one year, indicating that the accuracy of the three separate approaches depends on the time window length. The more extensive the time range, the greater the separation accuracy of the three methods. 

In summary, the proposed method is better than the wavelet-based and EEPI-based methods for the separation accuracy at different time scales. The proposed method has a more significant superiority with the shorter time window. With the expansion of the time range, the proposed approach will become more accurate. Overall, the separation error δ is small, demonstrating the high separation accuracy of the proposed method.

### 3.2. Anti-Noise Discussion

**Figure 11:**
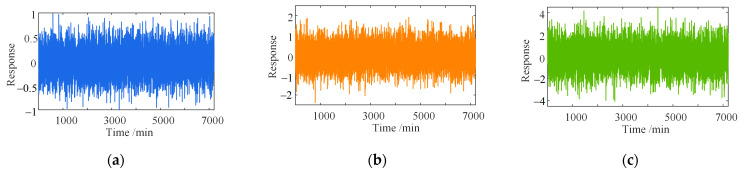
Different degrees of noise (five days): (**a**) $e_{p}=5\%$; (**b**) $e_{p}=10\%$; (**c**) $e_{p}=20\%$.

**Figure 12:**
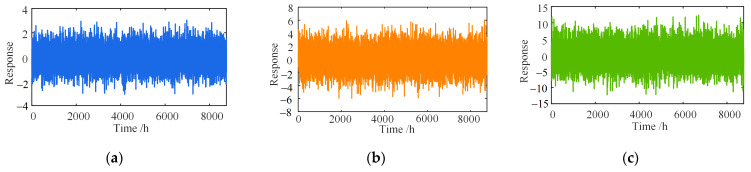
Different degrees of noise (one year): (**a**) $e_{p}=5\%$; (**b**) $e_{p}=10\%$; (**c**) $e_{p}=20\%$.

Noise intensities of 5%, 10%, and 20% were applied to the mixed signals in Section 3.1 to determine the effect of the different noise levels on the separation results. The noise is simulated by Equation (30) [43]. The different degrees of noise added in the different time scales are shown in Figure 11 and Figure 12, respectively.

(30) $$D_{E}=e_{p}\times \max(D_{T})\times \left(0.5\times N_{o}+0.5\times R_{o}\right)$$

where $e_{p}$ represents the intensity of noise, $N_{o}$ is a normal distribution with mean value 0 and variance 1, and $R_{o}$ is a uniform distribution on the interval [−1, 1].

**Figure 13:**
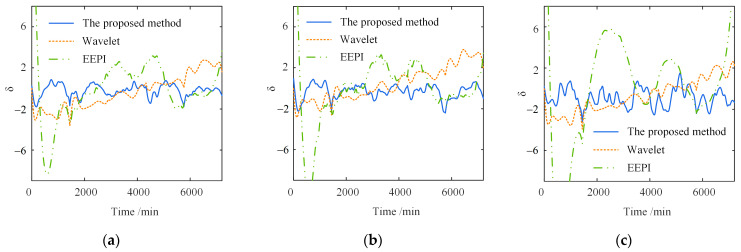
Separation error under different degrees of noise (five days): (**a**) $e_{p}=5\%$; (**b**) $e_{p}=10\%$; (**c**) $e_{p}=20\%$.

**Figure 14:**
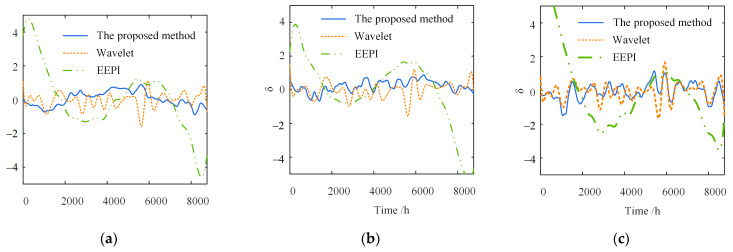
Separation error under different degrees of noise (one year): (**a**) $e_{p}=5\%$; (**b**) $e_{p}=10\%$; (**c**) $e_{p}=20\%$.

Figure 13 and Figure 14 show that the separation error δ of the proposed method and wavelet-based method change little under different time scales and degrees of noise. With the increase in the noise level, the error fluctuation degree of the two methods grows, but the error range is basically unchanged. However, with the increase in the noise level, the separation error of the EEPI-based method will grow gradually. When the intensity of the noise is more than 10%, the separation effect is poor, which is consistent with the conclusion in the literature [25]. Therefore, noise has no significant effect on the separation results of either the proposed method or the wavelet-based method. The separation result of the EEPI-based method is more susceptible to noise.

## 4. In Situ Measured Signal Discussion

### 4.1. Introduction of the Bridge and the Monitoring System

A heavy haul railway bridge of 96.75 m + 132.0 m + 96.75 m is a prestressed concrete continuous rigid-frame bridge. The main girder is a single box and single chamber section. The beam height at the fulcrum is 9.2 m, and the beam height at the mid-span and the end of the side span is 5.0 m. The lower edge of the beam changes according to the quadratic parabola, except that the middle fulcrum is 12.0 m, the middle span is 10.0 m, and the end of the side span is 35.7 m. The width of the top plate is 8.1 m, and the width of the bottom plate is 6.1 m. The thickness of the roof is 50 cm, the thickness of the bottom plate changes from 40 cm to 90 cm according to the quadratic parabola, and the thickness of the web changes from 45 cm to 90 cm according to the broken line.

**Figure 15:**
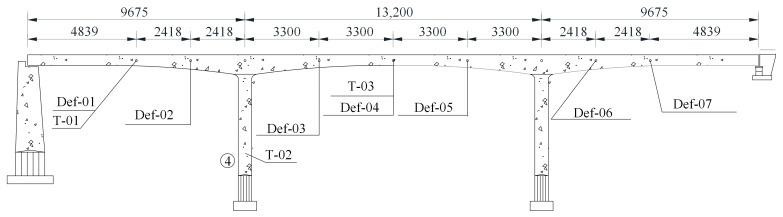
Longitudinal section layout of the bridge sensors/cm.

**Figure 16:**
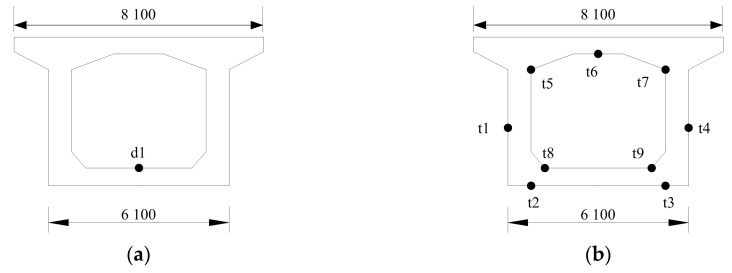
Cross-section layout of the bridge sensors/mm. (**a**) Deflection sensor; (**b**) temperature sensors.

The bridge is equipped with temperature sensors and vertical deflection sensors, and the frequency of the sensors is 1/600 Hz and 1 Hz, respectively. The sensor layout on the bridge is shown in Figure 15 and Figure 16.

### 4.2. Effective Temperature

The effective temperature is commonly used to describe the distribution of the temperature on a cross-section, offering crucial data for long-span bridge designs. The calculation of the temperature-induced response should adopt the effective temperature of the entire section rather than the individual temperature data obtained from any one sensor. It can be expressed as [30]

(31) $$T_{e}=\frac{\int E\alpha TdA}{\int E\alpha dA}$$

where *E* is Young’s modulus, α is the coefficient of the thermal expansion, *T* is the temperature, and *dA* is the differential area of the cross-section.

### 4.3. Temperature-Induced Response Separation and Correlation Analysis

**Figure 17:**
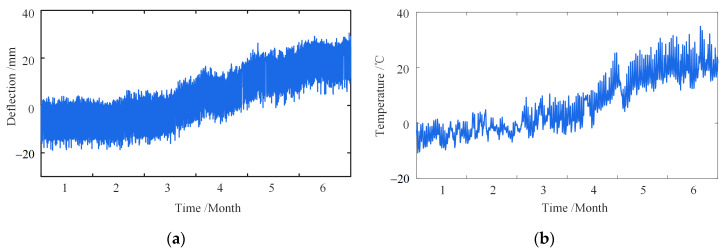
In situ measured signal of the bridge. (**a**) In situ measured deflection; (**b**) effective temperature.

Figure 17 shows the field-measured deflection and effective temperature of the Def-04 measuring points in the first half of 2020. As shown in Figure 17a, the deflection change is noticeable. The deflection is a gentle trend before late February, while from late February to late June, it shows a rapid upward trend. The trend of the temperature and deflection is the same, indicating a specific correlation between them.

**Figure 18:**
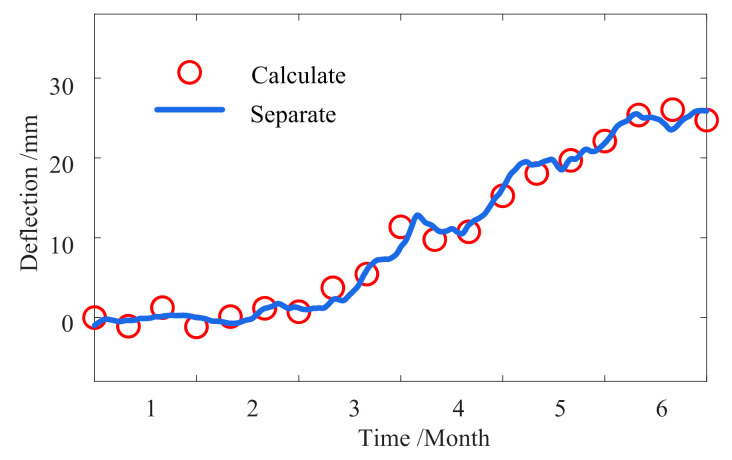
Comparison between the calculation results of the FEM and the separated deflection.

To explore the specific correlation, it is required to separate the temperature-induced response from the measured deflection. An FEM is established for the bridge by a space beam element in MIDAS, and each element is 1.5 m long. The bridge structural deflection is calculated using the maximum temperature difference every ten days as the system temperature input to MIDAS. The calculation deflection and the separation response are shown in Figure 18.

**Figure 19:**
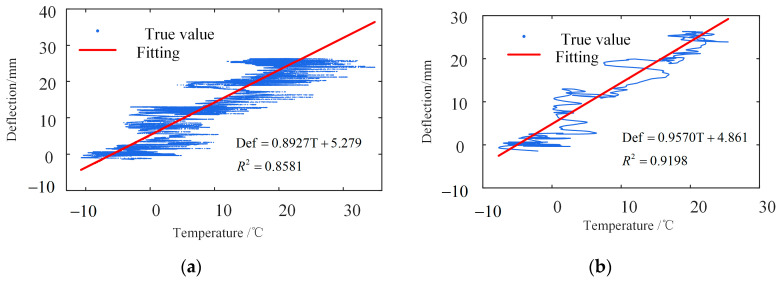
Correlation between separated deflection and temperature before and after filtering. (**a**) Temperature before filtering; (**b**) temperature after filtering.

Figure 18 shows that their trends and amplitudes are the same, which further verifies the rationality and reliability of the separation method. The separated deflection changes significantly within half a year, up to 30 mm, indicating that the temperature intensely affects the structure. The correlation coefficient is 0.8581 by a regression analysis of the measured temperature and deflection data. The fitting line and the measured values are shown in Figure 19a.

Figure 19a shows that most points are concentrated near a straight line. However, some points are far from the straight line due to measurement errors or other factors. The measured temperature data are filtered-out noise. The linear fitting is carried out, and the fitting line and the measured value are shown in Figure 19b. The correlation coefficient is 0.9198, and the correlation between them is increased by 7.2%. 

## 5. Conclusions

This paper presents an adaptive method of temperature-induced response separation using the local outlier correction and Savitzky–Golay convolution smoothing. The proposed method utilizes the LOF to transform the in situ measured data and optimizes the thresholds of the LOF using the AOHHO. The Savitzky–Golay convolution smoothing is employed to reduce the effect of the noise in the modified data. The proposed approach is compared with the wavelet-based and EEPI-based approaches to demonstrate its performance. Finally, the correlation between the temperature and structural response is analyzed, using the proposed method in engineering practice. The conclusions of this study are presented below: 

(1) The AOHHO algorithm has a faster convergence speed and stronger global optimization ability than the four metaheuristic algorithms of the AO, HHO, PSO, and WAO. The AOHHO maximizes the benefits of these two fundamental algorithms by retaining the exploration phase of the AO and the exploitation phase of the HHO.

(2) The proposed method has a high accuracy and is not sensitive to noise. The separation accuracy of the proposed approach in different time windows is better than the wavelet-based and EEPI-based methods. The maximum separation errors of the two methods are about 2.2 times and 5.1 times that of the proposed method, respectively. The shorter the time window, the greater the superiority of the proposed method. The proposed method is more accurate when the time window is extended. In addition, the proposed method expands the practical application range because the only structural response is required without the temperature distribution.

(3) A strong linear correlation exists between the temperature-induced deflection and the effective temperature, and the effects of long-term temperature variations on long-span prestressed concrete continuous rigid-frame bridges cannot be ignored. The deflection changes up to 30 mm within half a year. In addition, after filtering the measured temperature, the linear correlation between the effective temperature and the deflection is increased by 7.2%.

(4) The proposed method filters out the short-term effects from the structural responses. It has not been proved whether the separated response is entirely caused by the temperature, even if the temperature is usually the decisive factor. Further research is necessary to separate the response caused by the shrinkage, creep, and prestress from the measured signal. A promising area for future research is a structural damage diagnosis and safety warning using various separated responses.

## Figures and Tables

**Table 1:** Benchmark functions.

Function	Expression	Dimension	Range	Minimum Value
F1	$f(x)=\overset{n-1}{\underset{i=1}{\sum }}[100{\left(x_{i}^{2}-x_{i+1}^{20}\right)}^{2}+{\left(1-x_{i}\right)}^{2}]$	100	[−100, 100]	0
F2	$f(x)=\overset{n}{\underset{i=0}{\sum }}ix_{i}^{4}+random[0,1)$	30	[−1.28, 1.28]	0
F3	$\begin{array}{c} f(x)=\frac{\pi }{n}\left\{10\sin\left(\pi y_{1}\right)\right\}+\sum_{i=1}^{n-1}{\left(y_{i}-1\right)}^{2}\left[1+10\sin^{2}\left(\pi y_{i+1}\right)+\sum_{i=1}^{n}u\left(x_{i},10,100,4\right)\right],\;\\ \mathrm{where}\;y_{i}=1+\frac{x_{i}+1}{4},u\left(x_{i},a,k,m\right)\{\begin{array}{ll} K{\left(x_{i}-a\right)}^{m} & \;if\;x_{i}>a\\ 0 & -a<x_{i}\leq a\\ K{\left(-x_{i}-a\right)}^{m} & -a⩽x_{i} \end{array} \end{array}$	30	[−50, 50]	0
F4	$f(x)={\left(\frac{1}{500}+\sum_{j=1}^{25}\frac{1}{j+\overset{2}{\underset{i=1}{\sum }}}\left(x_{i}-a_{ij}\right)\right)}^{-1}$	2	[−65, 65]	1

**Table 2:** Various calculation parameters.

$\left|N_{k}\left(x\right)\right|\;$	*k*	*a*	*b*	*c*	*Q*
8	5	1	2.5	0.05	10

## Data Availability

The data presented in this study are available on request from the corresponding author.
